# Left ventricular twist abnormalities in patients with left ventricular non-compaction. A cardiovascular magnetic resonance study

**DOI:** 10.1186/1532-429X-14-S1-P143

**Published:** 2012-02-01

**Authors:** Karol Miszalski-Jamka, Michael Taylor, Jan Glowacki, Kan N Hor, Tomasz Miszalski-Jamka, Jaroslaw Rycaj, Tomasz Adamczyk, Radoslaw Kwiecinski, Jan Klys, Kathleen I Williams, Victoria M Huang, Ewa Kluczewska, Zbigniew Kalarus, Wojciech Mazur

**Affiliations:** 1 Department of Cardiology, Congenital Heart Disease and Electrotherapy, Silesian Center for Heart Disease, Zabrze, Poland; 2 Diagnostic Imaging Department, Silesian Center for Heart Disease, Zabrze, Poland; 3 The Heart Institute, Cincinnati Children’s Hospital Medical Center, Cincinnati, OH, USA; 4 The Heart and Vascular Center, The Christ Hospital, Cincinnati, OH, USA; 5 Center for Diagnosis, Prevention and Telemedicine, John Paul II Hospital, Cracow, Poland; 6 Department of Cardiology, Medical University of Silesia, Katowice, Poland; 7 Department of Radiology, Medical University of Silesia, Katowice, Poland

## Background

Current morphologic left ventricular (LV) non-compaction (LVNC) criteria seem to be still insufficient. The prevalence of borderline degree of LV hypertrabeculation is far more frequent than initially expected. LV twist and strain values are proposed as potential new functional parameters supplementing the classic morphologic LVNC criteria. The aim of this study was to assess diagnostic utility of LV twist and strain analysis in detecting regional myocardial dysfunction in patients with LVNC.

## Methods

Cardiovascular magnetic resonance (CMR) examinations were performed in 52 patients with LVNC and 28 healthy controls with LV hypertrabeculation. LVNC was diagnosed according to CMR diagnostic criteria published by Petersen and coworkers: a two-layered structure of the LV wall, with the end-diastolic ratio of non-compacted to compacted layer >2.3. LVNC patients with concomitant congenital heart disease, significant valvular disease or coronary artery disease were excluded from this study. Using a dedicated feature tracking software (Diogenes MRI, TomTec Imaging Systems, Munich, Germany) CMR cine-images were analyzed to measure LV rotation/twist and circumferential strain. Standard 17-segment model of LV was used. Twist and strain values below or above two standard deviations of the average values in controls were considered abnormal.

## Results

In 52 patients with LVNC mean number of affected LV segments was 8.7±3.0, mean maximal end-diastolic ratio of non-compacted to compacted layer was 2.9±0.9, mean trabeculated LV mass was 27.1±8.0% of the global LV mass. Patients with LVNC were divided into two groups: with LV ejection fraction (EF)≥50% (n=25) and EF<50% (n=27).

LV twist values in LVNC patients with EF<50% were significantly reduced compared with LVNC patients with EF≥50% (5.9±3.1 vs 12.4±5.2, P<0.00001). Mean LV twist values in LVNC patients with EF≥50% and controls were not statistically different. However, in 7 (28%) patients with LVNC with EF≥50%, LV twist values were paradoxically increased. LV global circumferential strain values were significantly reduced in LVNC patients with EF<50% compared with LVNC patients with EF≥50% (-10.5±5.1 vs -19.4±3.7, P<0.00001). However, LV global circumferential strain values were not significantly different between LVNC patients with EF≥50% and controls.

## Conclusions

LV twist is significantly reduced in LVNC patients with decreased EF. However, in some LVNC patients with preserved EF, LV twist is paradoxically increased as a possible compensatory mechanism to maintain LV output. LV circumferential strain values, although reduced in LVNC patients with decreased EF, are not useful to discriminate between LVNC patients with preserved EF and controls.

## Funding

Self-funded.

